# Implementation of routine genomic surveillance provided insights into a locally acquired outbreak caused by a rare clade of *Salmonella enterica* serovar Enteritidis in Queensland, Australia

**DOI:** 10.1099/mgen.0.001059

**Published:** 2023-07-17

**Authors:** Irani U. Rathnayake, Rikki M. A. Graham, Jo Bayliss, Megan Staples, Gino Micalizzi, Lawrence Ariotti, Leonie Cover, Brett Heron, Trudy Graham, Russell Stafford, Sally Rubenach, Andrew D'Addona, Amy V. Jennison

**Affiliations:** ^1^​ Public Health Microbiology, Forensic and Scientific Services, Queensland Department of Health, Coopers Plains, Queensland, Australia; ^2^​ OzFoodNet, Communicable Diseases Branch, Queensland Public Health and Scientific Services, Queensland Department of Health, Butterfield Street, Herston, Brisbane, Queensland, Australia; ^3^​ Health Surveillance, Tropical Public Health Services Cairns, Cairns and Hinterland Hospital and Health Service, Queensland Department of Health, Cairns, Queensland, Australia; ^4^​ Environmental Health, Tropical Public Health Services Cairns, Cairns and Hinterland Hospital and Health Service, Queensland Department of Health, Cairns, Queensland, Australia

**Keywords:** outbreak, Salmonella, whole-genome sequencing, single nucleotide polymorphism, core-genome multilocus sequence typing

## Abstract

Salmonellosis is a significant public health problem globally. In Australia, *Salmonella enterica* serovar Enteritidis is one of the main causes of salmonellosis. This study reports how the implementation of routine genetic surveillance of isolates from human *S*. Enteritidis cases enabled identification of the likely source of an outbreak that occurred in a remote town in Far North Queensland, Australia. This study included patient, food and water samples collected during an outbreak investigation. *S*. Enteritidis of the novel sequence type 5438 was isolated from all seven patient samples and one bore water sample but not any of the food samples. Both whole-genome single nucleotide polymorphism (SNP) and core-genome multilocus sequence typing analysis revealed that *S*. Enteritidis isolated from outbreak-related patient samples and the bore water isolates clustered together with fewer than five SNP differences and ten allelic differences. This genetic relatedness informed the outbreak response team around public health interventions and no further cases were identified post-treatment of the bore water. This disease cluster was identified through the routine sequencing of *S*. Enteritidis performed by the state public health laboratory in an actionable time frame. Additionally, genomic surveillance captured a case with unknown epidemiological links to the affected community, ruled out a simultaneous outbreak in an adjacent state as the source and provided evidence for the likely source preventing further transmission. Therefore, this report provides compelling support for the implementation of whole-genome sequencing based genotyping methods in public health microbiology laboratories for better outbreak detection and management.

## Data Summary

All sequences generated in the current investigation have been uploaded to the European Nucleotide Archive (ENA) under Bioproject PRJEB57403.

**Impact Statement**

Whole-genome sequencing (WGS)-based genotyping methods facilitated the confirmation and investigation of an outbreak caused by a novel sequence type of *S*. Enteritidis in a remote Far North Queensland region. Furthermore, it provided evidence that the source of outbreak was untreated water sourced from a private bore. The routine implementation of WGS-based genotyping methods for S. Enteritidis in the public health microbiology laboratory for Queensland, Australia enabled the timely outbreak detection, case attribution, and guidance for public health interventions, which prevented the further spread of the outbreak. This study highlights the feasibility of rapid and robust WGS-based genotyping methods for both routine pathogen surveillance and deployment in outbreak investigations for public health microbiology laboratories.

## Introduction

*Salmonella* infection is one of the major public health concerns globally causing gastroenteritis, which can lead to complications such as bacteraemia [1, 2]. *Salmonella enterica* can be transmitted through food, water, the environment, animals and people [3]. According to Pearce *et al*., *S*. Enteritidis is the major single cause of *Salmonella* infection worldwide and accounts for 40–60 % of human cases [2]. In Australia, *S*. Typhimurium is the major cause of salmonellosis, followed by *S*. Enteritidis. Between 2009 and 2018, there were, on average, 825 notified cases of *S*. Enteritidis each year, with almost one quarter of these notified from Queensland [4]

Although the annual notification rate of *S*. Enteritidis infection in Australia between 2009 and 2018 remained relatively stable, the incidence in Queensland steadily increased from 2.4 to 4.0 cases/100 000 population during this same time period [4]. Rapid and accurate identification and typing of pathogens play an essential role in effective surveillance and outbreak investigation, especially for monomorphic bacteria such as *S. Enteritidis* [5, 6]. Conventional typing methods do not always have sufficient granularity and robustness to define strains unequivocally, and adequate epidemiological data are not always available to establish links between patients and the source of outbreak [7]. However, previous studies have demonstrated that whole-genome single nucleotide polymorphism (WG-SNP) based cluster analysis provides the resolution and accuracy required for surveillance and outbreak investigations of *Salmonella enterica* [6, 8].

The majority of *S*. Enteritidis strains identified in Australia are acquired overseas [9]. However, a comparative genomics study conducted by [10], using WG-SNP based cluster analysis revealed the existence of three distinct *S*. Enteritidis clades; A, B and C detected among isolates from Queensland, Australia, where clade B belonged to the previously identified global lineage but the two other clades; A and C appeared to be associated with acquisition in Australia [10].

An alternative approach to SNP typing; core-genome multilocus sequence typing (cgMLST), measures the genetic relatedness of isolates through sequence comparison across a defined set of loci, and has previously been used as a tool for *Salmonella* outbreak investigations [11].

In 2017, the Queensland public health reference laboratory implemented routine whole-genome sequencing (WGS) with clustering reported on all *S*. Enteritidis cases diagnosed in the state. In January 2019, the laboratory identified a genomic cluster of seven geographically clustered cases of *S*. Enteritidis from a remote Far North Queensland region with gastroenteritis symptoms including diarrhoea, abdominal pain, vomiting, fever and blood in stool. The cluster was caused by a strain belonging to clade C, which is relatively rare compared to the other *S*. Enteritidis clades circulating in Queensland. An outbreak investigation was instigated, and genomics was central to identifying cases and assisting in identifying the source of the outbreak.

## Methods

### Case identification and sample processing

A case was defined as any person who was a resident or visitor to the affected region between 1 December 2018 and 31 January 2019, who was notified with a *S*. Enteritidis infection. Those cases were subsequently confirmed as falling into clade C on phylogenetic analysis.

Following advice from the public health laboratory that a cluster of highly related cases of *S*. Enteritidis had been detected in a remote Far North Queensland region a multidisciplinary outbreak management team was established by the Public Health Unit to investigate the cluster. The remote location of the town and extreme weather events presented significant logistical challenges for the investigating team, with three separate field trips required to investigate the potential sources of infection across multiple locations in the region.

A standard hypothesis-generating questionnaire was administered to cases to collect clinical information, travel and food histories and environmental exposures during the week before onset of illness. Initial case interviews identified three stores selling food as possible sources. During the inspection of one of the identified stores it was noted that the bore water was used in the preparation of food sold and the UV treatment of a bore water source was not functioning. That bore water source services only that particular store and no other businesses or residential houses were using it as a water supply. Three bore water samples were collected from that store for further analysis to determine or exclude as a potential source of infection. In addition, 31 food samples from identified sites were collected and sent to the Public Health Microbiology Laboratory, Queensland Health Forensic and Scientific Services (QHFSS) for analysis. Eggs and chicken meat were collected so as to exclude them as a potential source of infection. Salads were sampled as bore water was used in the store to wash vegetables.

The bore water samples were analysed at a private laboratory in the region to meet the recommended transportation time of less than 24 h. Two *Salmonella* isolates from the bore water were sent by the private laboratory to QHFSS for further typing. Seven Salmonella isolates from patients were also referred by diagnostics laboratories to QHFSS for further typing. All isolates received at QHFSS were serologically classified according to White–Kauffmann–LeMinor scheme, as recommended by the WHO Collaborating Centre for Reference and Research on *Salmonella* [12].

The test portion and initial suspension of the food samples were prepared and incubated at 37° for 18–24 h according to Australian Standard AS 5013.10 : 2022 [13]. Incubated suspensions were screened for *Salmonella* spp. using the BAX Q7 DNA-based detection system by Hygiena. Presumptive positive samples were cultured according to AS 5013.10 : 2022, confirmed using the Bruker matrix-assisted laser desorption/ionization-time of flight mass spectrometer and serotyped.

All *S*. Enteritidis isolates with a link to the outbreak were subjected to WGS (Table 1).

**Table 1.** Isolates analysed for the outbreak investigation in a remote community in North Queensland at QHFSS

Sample type	ID	Description	Detection of *S*. Enteritidis	SNP clade	MLST
**Human**	M190020	Isolate from faeces	Detected	C	5438
	M190123	Isolate from faeces	Detected	C	5438
	M190151	Isolate from faeces	Detected	C	5438
	M190196	Isolate from faeces	Detected	C	5438
	M190395	Isolate from faeces	Detected	C	5438
	M190396	Isolate from faeces	Detected	C	5438
	M190977	Isolate from faeces	Detected	C	5438
**Water**	M1942 NUT^*^	Isolate from bore water	Detected	C	5438
	M1942 PURP^*^	Isolate from bore water	Detected	C	5438

*Different colonies from the same isolate.

### WGS of the bacterial strains

DNA was extracted from *S*. Enteritidis isolates grown overnight at 37 °C on Xylose Lysine Deoxycholate agar (XLD), using the QiaSymphony DSP DNA Mini kit (Qiagen, Germany) according to the manufacturer’s instructions. DNA concentrations were measured using Quant-iT High-Sensitivity dsDNA Assay Kit as per manufacturer’s instructions (Thermo Fisher Scientific, USA) and normalized to 0.3 ng µl^–1^ and stored at −20 °C prior to library preparation.

Libraries were prepared with a Nextera XT DNA sample preparation kit (Illumina, USA), and sequenced on the NextSeq500 with the 500 Mid Output v2.5 kit (300 cycles) (Illumina, USA) according to the manufacturer’s instructions.

### Bioinformatic analysis

Generated sequences were quality trimmed using Trimmomatic v0.36 [14]. Trimmed sequences were *de novo* assembled into contigs using the SPAdes assembler V3.12.0 (https://github.com/ablab/spades) [15]. MLST analysis was performed on the contigs using145 the *S. enterica* MLST scheme in Enterobase (https://enterobase.warwick.ac.uk/) [16].

The Snippy pipeline V4.3.6 was used to identify SNPs using S. *Enteritidis* P125109 (GenBank accession NC_011294) as the reference genome (https://github.com/tseemann/snippy). Core SNPs from each sample and an additional 233 historical isolates were aligned using Snippy core, which is also part of the Snippy package. These 233 historical isolates were sequenced by QHFSS from 2018 to 2019 January and included as context isolates for the outbreak investigation. A maximum-likelihood tree was generated from the SNP alignment, with the RAxML wrapper in Geneious R11 keeping 100 rapid bootstrap inferences and using the substitution model GTRGAMMA (general time reversible model of nucleotide substitution with the GAMMA model of rate heterogeneity) (Biomatters, New Zealand) [17]. All the outbreak isolates belonged to clade C. Sequences were then re-analysed in the context of only clade C isolates using a clade C specific reference sequence, M1510738 (GenBank accession number CANERU000000000.1). The SNP distances were determined using the distance matrix generated in Geneious R11 using core SNPs' alignment and maximum-likelihood tree was generated for clade C as described before using Geneious R11. Historical epidemiologically confirmed clusters had previously been used to determine expected SNP ranges for closely related isolates to form a cluster, and a SNP distance of five between isolates was considered the threshold to define a cluster in the QHFSS laboratory [18].

cgMLST analysis was performed using the *S. enterica* cgMLST scheme developed by Enterobase; cgMLSTV2 (https://enterobase.warwick.ac.uk/) to validate the SNP findings. A Grape tree was generated within the Enterobase scheme using Hierarchical Clustering of CgMLST (HierCC). HC 10 was used as the cluster threshold where all isolates connect to each other in a single-linkage clustering tree with no more than ten allelic differences.

MLVA (multiple locus variable-number tandem repeat analysis) *in silico* typing resource for salmonella strains (MISTReSS, https://github.com/Papos92/MISTReSS#mistress-mlva-in-silico-typing-resource-for-salmonella-strains) [19] was performed to compare the results with SNP typing and cgMLST typing.

## Results and discussion

A cluster of cases in a remote Far North Queensland Region was investigated in this study. The causative agent of the outbreak was identified as *S*. Enteritidis. Seven patient isolates and two bore water isolates were confirmed as *S*. Enteritidis.

A total of 31 samples of cooked and raw foods was collected from epidemiologically linked food stores in the region, including from a food store where issues with the treatment of bore water was identified. *S. Enteritidis* was not detected in any of the submitted foods, which may have been due to a number of reasons including final bacterial load on the foods being below culture limits of detection and that most of the collected products had undergone processing steps like heating.

Sequencing and MLST analysis of isolates revealed that the *S. Enteritidis* responsible for the outbreak was a new sequence type (ST) harbouring a novel *hisD* gene allele. The sequence was submitted to the EnteroBase database (https://enterobase.warwick.ac.uk/species/index/senterica) and assigned a new ST number; ST5438. While the strain associated with cases and the bore water samples was determined to be the same novel sequence type, the limited information on sequence types of *S.* Enteritidis from environmental samples in Far North Queensland meant that higher resolution SNP-based typing via genomics was performed to further investigate the relatedness between the isolates.

**Fig. 1.**
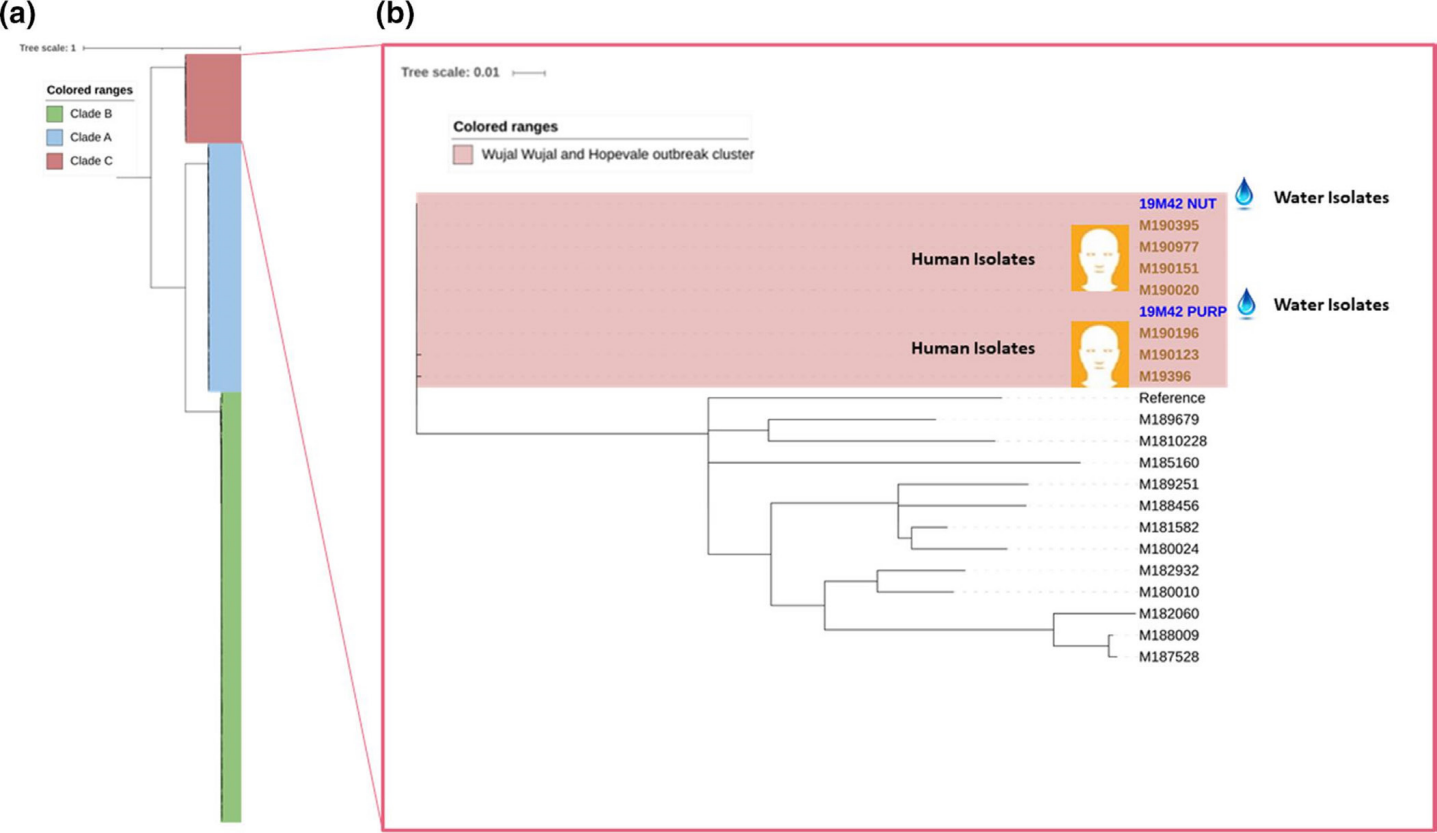
Maximum-likelihood phylogeny of outbreak related *S*. Enteritidis compared to other Queensland (QLD) *S*. Enteritidis. (a) Phylogenetic maximum-likelihood tree built using SNP differences between QLD *S*. Enteritidis isolates. This consist of 66, 155 and 21 isolates from clade A, clade B and clade C, respectively. Coloured ranges, blue, green and red, indicate the clade A, clade B and clade C, respectively. The scale bar corresponds to nucleotide substitutions per site. (b) Phylogenetic maximum-likelihood tree built using SNP differences between clade C QLD *S*. Enteritidis isolates, which were analysed separately to obtain the better discrimination. Red coloured range indicates the outbreak cluster. Isolates in blue and brown are isolated from water and humans, respectively. The scale bar corresponds to nucleotide substitutions per site.

SNPs can be highly informative markers and SNP-based analyses have been successful in resolving outbreaks previously [2, 20, 21]. Maximum-likelihood phylogenies generated by SNP alignment of outbreak-related sequences and 233 previously snipped samples revealed that the *S*. Enteritidis strains grouped into three distinct clades, which consist of 66, 155 and 21 isolates from clade A, clade B, and clade C, respectively (Fig. 1a). This result is consistent with a previous study by Graham *et al*. [10]. The outbreak sequences clustered into clade C, and the SNP tree for clade C is shown in Fig. 1b. This clade has previously been associated with locally acquired infections. As seen in Fig. 1b, the outbreak patient isolates cluster with the bore water isolate, where 0–1 SNP difference were identified between sequences. Other sequences included for context had >100 SNP difference to the outbreak isolates. These isolates showed a high degree of genetic similarity and were more closely related to each other than to other clade C isolates included in the analysis for context.

**Fig. 2.**
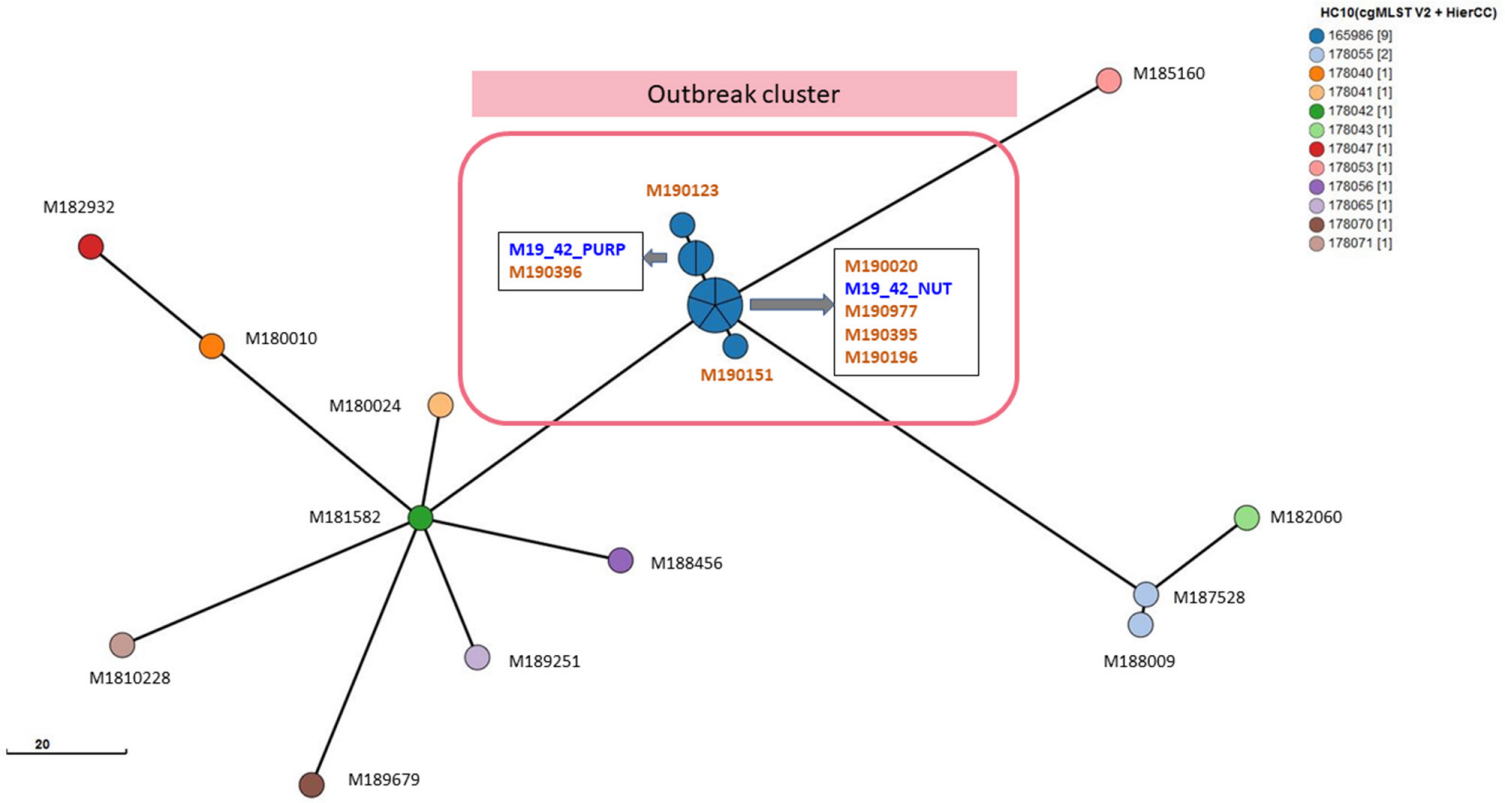
HierCC analysis of clade C. Different colours indicate the cgMLST clusters where all isolates connect to each other in a single-linkage clustering tree with no more than ten allelic differences (HC 10). Red coloured rectangle indicates the outbreak clusters. Isolates in blue and brown are isolated from water and humans, respectively. The scale bar indicates the number of cgMLST allelic differences.

SNP findings were in concordance with cgMLST findings, where all outbreak-related *S*. Enteritidis isolates clustered together with fewer than ten allelic differences in the cluster and they all belonged to the same clonal complex 165 986 (Fig. 2). According to MISTReSS analysis, clade C (outbreak-related clade) and clade A did not provide full MLVA profiles where some repeats were missing. However, our clade B provided the full profile. This may be due to clade A and C consisting of locally acquired isolates where clade B is typically associated with overseas acquired isolates. These results suggested that the SNP and cgMLST typing methods are universally applicable platforms regardless of the origin of the isolate. However, further validation of MISTReSS is required to better understand the typeability for clade A and C strains.

One of the patients (M190123) in the outbreak cluster was an overseas resident travelling through the area and was identified incidentally through the routine genomic surveillance of all Queensland cases. Once highlighted as clustering with the outbreak, subsequent public health follow up confirmed that the case had visited the affected region regularly at the time of the outbreak. Routine testing of *S*. Enteritidis isolates using WGS, and SNP analysis was able to link this case to the outbreak, something that would have been missed had the outbreak investigation focussed solely on residents of the region affected.

At the time of this disease cluster, a large *S.* Enteritidis outbreak was occurring an adjacent Australian state that had emerged in May 2018 was still under investigation [22]. Rapid genomic comparison was used to rule out a link between this Far North Queensland cluster and the interstate outbreak, saving public health efforts and resources from further exploring that hypothesis.

Furthermore, according to the SNP and cgMLST results, sequences from the outbreak-related patient isolates formed a monophyletic group with the bore water isolates. This evidence along with the epidemiological data from patients’ food consumption history and the findings of environmental health inspections of the site indicated that the *S*. Enteritidis strain that caused the outbreak was transmitted via bore water used at a local store. The Local Government Authority undertook the actions required by the Public Health Unit to appropriately treat the bore water. There were no further detections of *S*. Enteritidis in subsequent water samples submitted and no further *S.* Enteritidis cases reported from the region or identified in the ongoing genomics surveillance.

Genetic variation between *S*. Enteritidis strains is very limited. Widely used genotyping methods including MLVA and PFGE often do not provide satisfactory discriminatory power to differentiate between outbreak and sporadic samples in this serovar, which limits the utility of using these subtyping methods [23–25]. Increasingly, public health laboratories are replacing traditional typing methods, such as phage typing or PFGE and MLVA, with genomics-based analysis, which has become an important first-line public health microbiological method utilized for *Mycobacterium tuberculosis *, Shiga toxin-producing *Escherichia coli* and *Shigella* species in outbreak investigation [26]. This transition is occurring due to the compelling advantages WGS offers through increased typing discrimination, transportable data, pathogen agnostic methodology and cost effectiveness compared to traditional methods. As demonstrated in this outbreak, WGS was able to produce the required discriminatory power not only to tease out an *S*. Enteritidis outbreak from routine surveillance sequence data, allow an incidental case to be linked to the outbreak and confirmed the transmission source, all in sufficient real time to contribute this valuable guidance to the outbreak investigation and response.

This outbreak investigation demonstrates the value and practical feasibility of routine application of rapid, robust and affordable WGS technologies and associated bioinformatics to support the role of public health laboratories in ongoing disease surveillance and outbreak response.
