# Corrigendum: ‘Implementation of routine genomic surveillance provided insights into a locally acquired outbreak caused by a rare clade of *Salmonella enterica* serovar Enteritidis in Queensland, Australia’

**DOI:** 10.1099/mgen.0.001096

**Published:** 2023-08-17

**Authors:** Irani U. Rathnayake, Rikki M. A. Graham, Jo Bayliss, Megan Staples, Gino Micalizzi, Lawrence Ariotti, Leonie Cover, Brett Heron, Trudy Graham, Russell Stafford, Sally Rubenach, Andrew D'Addona, Amy V. Jennison

**Affiliations:** ^1^​ Public Health Microbiology, Forensic and Scientific Services, Queensland Department of Health, Coopers Plains, Queensland, Australia; ^2^​ OzFoodNet, Communicable Diseases Branch, Queensland Public Health and Scientific Services, Queensland Department of Health, Butterfield Street, Herston, Brisbane, Queensland, Australia; ^3^​ Health Surveillance, Tropical Public Health Services Cairns, Cairns and Hinterland Hospital and Health Service, Queensland Department of Health, Cairns, Queensland, Australia; ^4^​ Environmental Health, Tropical Public Health Services Cairns, Cairns and Hinterland Hospital and Health Service, Queensland Department of Health, Cairns, Queensland, Australia

## Full-Text

In the published version of the article, Fig. 1 identified two regions by name, which were deemed to be sensitive information. The corrected figure is shown below and has also been updated in the original published paper.

**Fig. 1.**
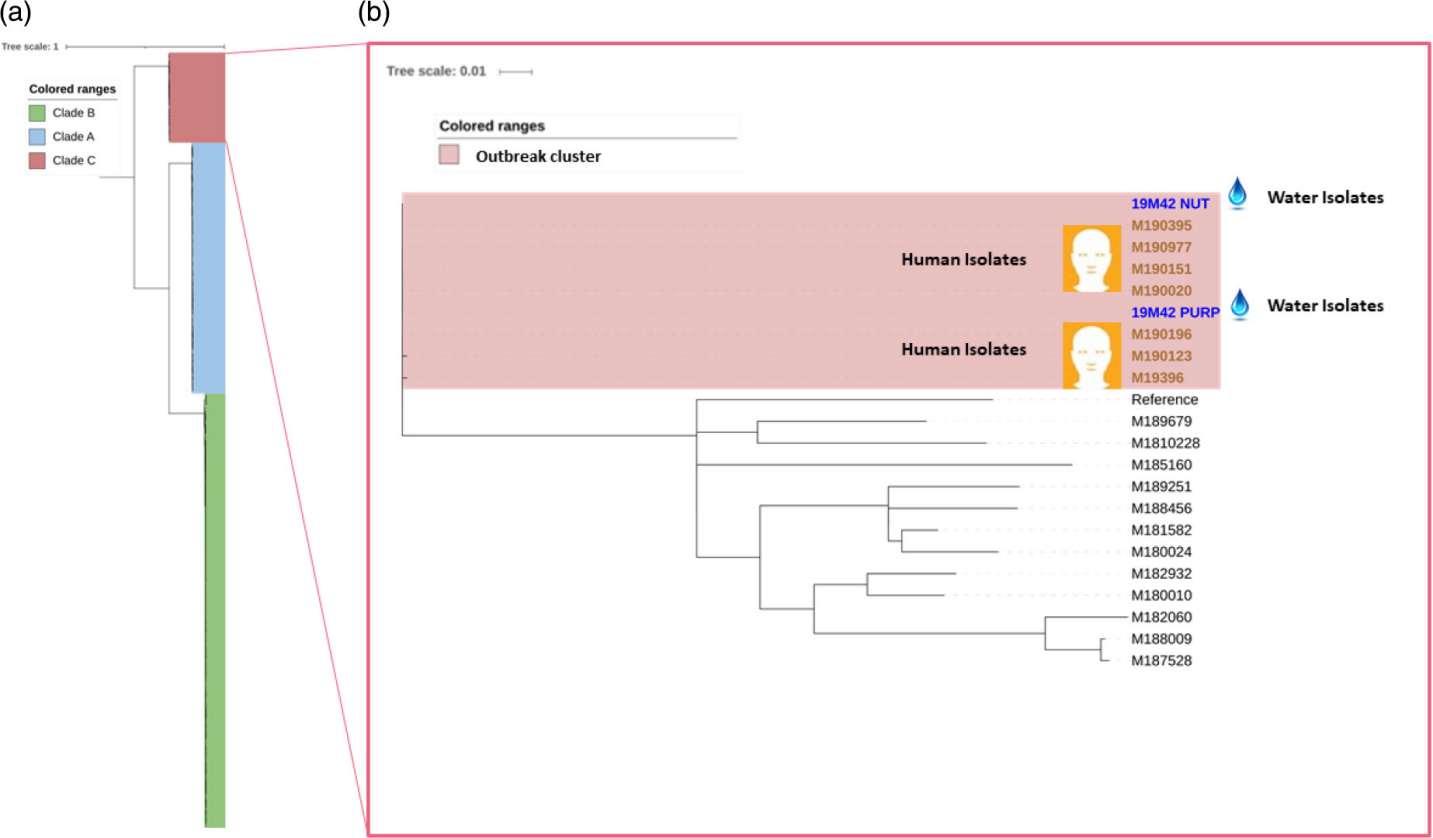
Maximum-likelihood phylogeny of outbreak related S. Enteritidis compared to other Queensland (QLD) S. Enteritidis. (**a**) Phylogenetic maximum-likelihood tree built using SNP differences between QLD S. Enteritidis isolates. This consist of 66, 155 and 21 isolates from clade A, clade B and clade C, respectively. Coloured ranges, blue, green and red, indicate the clade A, clade B and clade C, respectively. The scale bar corresponds to nucleotide substitutions per site. (**b**) Phylogenetic maximum-likelihood tree built using SNP differences between clade C QLD S. Enteritidis isolates, which were analysed separately to obtain the better discrimination. Red coloured range indicates the outbreak cluster. Isolates in blue and brown are isolated from water and humans, respectively. The scale bar corresponds to nucleotide substitutions per site.

The authors apologise for any inconvenience caused.

